# Pandemic (H1N1) 2009 Reinfection, Chile

**DOI:** 10.3201/eid1601.091420

**Published:** 2010-01

**Authors:** Carlos M. Perez, Marcela Ferres, Jaime A. Labarca

**Affiliations:** Pontificia Universidad Catolica de Chile, Santiago, Chile

**Keywords:** Influenza, pandemic (H1N1) 2009, reinfection, pandemic, subtype H1N1, viruses, Chile, letter

**To the Editor:** Since March 2009, influenza A pandemic (H1N1) 2009 has spread worldwide (*1*), and in South America, Chile was 1 of the countries most affected by the pandemic, with 21.4 cases among every 1,000 persons. Treatment guidelines in Chile recommended antiviral drug treatment with oseltamivir or zanamivir for 5 days for all patients with confirmed or suspected virus subtype H1N1 infection (*2*). In persons with seasonal influenza, specific antibody responses reach peak titers by 4 weeks after infection and confer protection against the infecting strain and closely related strains (*3*). Reinfection is rarely seen in nonpandemic influenza A. We report on 3 patients with confirmed influenza A pandemic (H1N1) 2009 reinfection after successful treatment with oseltamivir.

Patient 1, a healthy 14-year-old girl, had fever, sore throat, and nasal congestion on clinical examination. Pandemic (H1N1) 2009 infection was diagnosed by viral culture and confirmed by PCR specific for subtype H1N1 (LightMix Kit Inf A swine H1; TIB MOLBIOL, Berlin, Germany, for Roche Diagnostic, Indianapolis, IN, Light Cycler 2.0 instrument). The patient received oseltamivir, and symptoms resolved 48 hours after treatment. Twenty days later, fever, muscle aches, and vomiting developed in the patient. Influenza A virus was isolated by viral culture. The patient received a preliminary diagnosis of seasonal influenza and was treated with amantadine. She recovered from the infection before PCR results confirmed it was caused by pandemic (H1N1) 2009 virus.

Patient 2, a 62-year-old woman, experienced a high fever, cough, and nasal congestion during a prolonged hospitalization for bowel resection after intestinal ischemia. Pandemic (H1N1) 2009 was confirmed by PCR and viral culture. Oseltamivir was administered 5 days after the onset of symptoms, and the symptoms resolved within the following 5 days. The patient had a new episode of fever, productive cough, and bronchial obstruction 2 weeks later while still hospitalized. Culture results were positive for influenza, and PCR results were positive for pandemic (H1N1) 2009. The patient was again treated with oseltamivir, and PCR results were negative for influenza after 48 hours of antiviral treatment.

Patient 3, a previously healthy 38-year-old man, underwent mitral and aortic valve replacement while hospitalized for acute mitral and aortic endocarditis due to *Staphylococcus aureus*. Eleven days after surgery, he had a sore throat, nasal congestion, cough, and low-grade fever. PCR test results were positive for pandemic (H1N1) 2009. The patient received oseltamivir, and respiratory symptoms resolved within 5 days. He was discharged from the hospital but was readmitted 18 days later with nasal congestion, cough, and high fever. PCR results were again positive for pandemic (H1N1) 2009, and the patient was successfully treated with oseltamivir.

Patient 2 and probably patient 3 acquired their infections while hospitalized, suggesting potential nosocomial transmission. No other respiratory viruses were found in any of these patients. The viral isolates were all tested (LightMix for detection of influenza A virus oseltamivir resistance [H274Y]; TIB MOLBIOL) for possible resistance to oseltamivir, but none had the resistance-implicated H274Y mutation in the neuraminidase gene

Shedding of seasonal influenza A virus ceases within 5–7 days during natural infection and during infections treated with neuraminidase inhibitors (*4*). Although clearing of virus after the first infection was not documented in the 3 patients described here, it is unlikely that virus persisted between the 2 episodes of influenza since each of the patients fully recovered after specific antiviral drug treatment. However, we cannot rule out that patient 2 may have never cleared the virus due to her immune suppression.

As described by mathematical modeling (*5*), the 3 patients described were susceptible to reinfection with pandemic (H1N1) 2009 due to the high rate of community infection and to their incomplete immunologic protection within the period of reexposure. During the current pandemic of influenza subtype H1N1, healthcare workers and patients should be aware that symptomatic reinfection might occur after a first episode of infection.
